# A Role for TLR4 in *Clostridium difficile* Infection and the Recognition of Surface Layer Proteins

**DOI:** 10.1371/journal.ppat.1002076

**Published:** 2011-06-30

**Authors:** Anthony Ryan, Mark Lynch, Sinead M. Smith, Sylvie Amu, Hendrik J. Nel, Claire E. McCoy, Jennifer K. Dowling, Eve Draper, Vincent O'Reilly, Ciara McCarthy, Julie O'Brien, Déirdre Ní Eidhin, Mary J. O'Connell, Brian Keogh, Charles O. Morton, Thomas R. Rogers, Padraic G. Fallon, Luke A. O'Neill, Dermot Kelleher, Christine E. Loscher

**Affiliations:** 1 Immunomodulation Research Group, School of Biotechnology, Dublin City University, Ireland; 2 Department of Clinical Medicine and Institute of Molecular Medicine, Trinity Centre for Health Sciences, Trinity College Dublin, St James's Hospital, Dublin, Ireland; 3 School of Biochemistry and Immunology, Trinity College, Dublin, Ireland; 4 Molecular Evolution Group, School of Biotechnology, Dublin City University, Ireland; 5 Department of Clinical Microbiology, St James Hospital, Trinity College, Dublin, Ireland; Stanford University, United States of America

## Abstract

*Clostridium difficile* is the etiological agent of antibiotic-associated diarrhoea (AAD) and pseudomembranous colitis in humans. The role of the surface layer proteins (SLPs) in this disease has not yet been fully explored. The aim of this study was to investigate a role for SLPs in the recognition of *C. difficile* and the subsequent activation of the immune system. Bone marrow derived dendritic cells (DCs) exposed to SLPs were assessed for production of inflammatory cytokines, expression of cell surface markers and their ability to generate T helper (Th) cell responses. DCs isolated from C3H/HeN and C3H/HeJ mice were used in order to examine whether SLPs are recognised by TLR4. The role of TLR4 in infection was examined in TLR4-deficient mice. SLPs induced maturation of DCs characterised by production of IL-12, TNFα and IL-10 and expression of MHC class II, CD40, CD80 and CD86. Furthermore, SLP-activated DCs generated Th cells producing IFNγ and IL-17. SLPs were unable to activate DCs isolated from TLR4-mutant C3H/HeJ mice and failed to induce a subsequent Th cell response. TLR4^−/−^ and Myd88^−/−^, but not TRIF^−/−^ mice were more susceptible than wild-type mice to *C. difficile* infection. Furthermore, SLPs activated NFκB, but not IRF3, downstream of TLR4. Our results indicate that SLPs isolated from *C. difficile* can activate innate and adaptive immunity and that these effects are mediated by TLR4, with TLR4 having a functional role in experimental *C. difficile* infection. This suggests an important role for SLPs in the recognition of *C. difficile* by the immune system.

## Author Summary

*Clostridium difficile* is the leading cause of antibiotic-associated diarrhoea among hospital patients and in severe cases can cause pseudomembranous colitis and even death. There is currently limited information regarding how this pathogen is recognised by the immune system and the key mechanisms necessary for clearance of the pathogen. *C. difficile* expresses a paracrystalline surface protein array, termed an S-layer, composed of surface layer proteins (SLPs). Their location on the outer surface of the bacteria suggests that they may be involved in immune recognition of the pathogen. In this study we demonstrate that these SLPs are recognised by toll-like receptor 4 (TLR4). Activation of TLR4 by SLPs resulted in maturation of dendritic cells and subsequent activation of T helper cell responses which are known to be important in clearance of pathogens. Furthermore, using a murine model of *C. difficile* infection we show that mice display increased severity of infection in the absence of TLR4. This is the first study to demonstrate a role for TLR4 in infection associated with *C. difficile* and suggests an important role for SLPs in the generation of the immune response necessary for clearance of this bacterium.

## Introduction

*Clostridium difficile* is a Gram-positive spore-forming intestinal pathogen. It is the leading cause of nosocomial antibiotic-associated diarrhoea among hospital patients and in severe cases can cause pseudomembranous colitis and even death [1], [2]. The pathogenesis of *C. difficile* has been attributed to the two major toxins that the bacterium produces [3], [4]; however, there is currently limited information regarding the recognition of this pathogen by the immune system and the immune response elicited following exposure to this organism. This may be due to the fact that this organism does not produce lipopolysaccharide and therefore has been less well studied than other gastrointestinal pathogens.

*C. difficile*, along with a number of other bacteria, expresses a paracrystalline surface protein array, termed an S-layer, composed of surface layer proteins (SLPs) [5]. Two surface layer proteins termed high molecular weight (HMW) and low molecular weight (LMW) SLPs, form a crystalline regular array that covers the surface of the bacterium. SLPs are known to have a role in binding of *C. difficile* in the gastrointestinal tract however they may also have other roles [6]. There is now clear evidence that these proteins are important components of *C. difficile* [7], and S-layers have previously been described as virulence factors for other bacteria such as *Campylobacter fetus* and *Aeromonas salmonicida* [8], [9]. Their location on the outer surface of the bacteria suggests that they may be involved in immune recognition of the pathogen.

Pathogen recognition involves a group of pattern recognition receptors expressed on immune cells called toll-like receptors (TLRs) which allow cells of the innate immune system, such as dendritic cells (DCs), to detect conserved patterns of molecules on pathogens [10]. Several studies have highlighted the importance of TLR4 in a number of bacterial infections. For example, the recognition of *Mycobacterium tuberculosis*, a Gram-positive bacterium, by TLR4 is critical for elimination of the pathogen and containment of the infection to the lungs [11]. Activation of TLR4 initiates downstream signalling which in turn activates nuclear factor kappa beta (NF-κB) and interferon regulatory factor 3 (IRF3) via myeloid differentiation factor 88 (MyD88) -dependant and -independent pathways, respectively [12], [13]. Activation of the MyD88 dependant pathway is mainly an event initiated at the plasma membrane while induction of IRF via the MyD88-independent pathway is dependant on the endocyotosis of TLR4 and requires the presence of CD14 and subsequently TIR-domain-containing adapter-inducing interferon-β (TRIF) [14], [15].

When triggered, TLRs induce strong immune and inflammatory responses, characterised by production of inflammatory cytokines and subsequent activation of T helper (Th) cells [16]. The maturation of DCs following activation is characterized by the production of cytokines and changes in the expression of cell surface markers. It is now well established that production of IL-12 promotes Th1 differentiation, IL-4 induces Th2 cells, while IL-23, IL-6 and IL-1β production by DCs is important in generating Th17 cells [17], [18]. The importance of Th1 and Th17 cells are well recognised in bacterial clearance [19].

In the present study we tested the hypothesis that SLPs isolated from *C. difficile* are important for recognition of the pathogen and examined whether recognition of SLPs was mediated by TLR4. We report that SLPs induce DC maturation and have the ability to subsequently generate Th1 and Th17 responses via TLR4. Furthermore, we provide evidence that SLPs activate NFκB, but not IRF3, downstream of TLR4. Finally, we show that TLR4 has a functional role in experimental *C. difficile* infection. This is the first study to report a mechanism of recognition of *C. difficile* by the innate immune system, and suggests that they are important for activating the immune system and subsequent clearance of the pathogen.

## Materials and Methods

### Animals and materials

BALB/c mice, C3H/HeN and C3H/HeJ mice were purchased from Harlan (U.K.) and were used at 10–14 wk of age. TLR2-deficient (^−/−^) [20], TLR4^−/−^ [21], MyD88^−/−^ [22] and TRIF^−/−^ [23], all on a C57BL/6J background, were used in *C. difficile* infection studies. Animals were housed in a licensed bioresource facility (Dublin City University or Trinity College Dublin) and had *ad libitum* access to animal chow and water.

### Ethics statement

All animal procedures were carried out in accordance with Department of Health and Children Ireland regulations and performed under animal license number B100/3250. All animal protocols received ethical approval from the Trinity College Dublin Bioresources Ethics Committee. *C. difficile* infected animals were weighed daily and any mice that became moribund, <15% loss in body weight, were humanely killed.

### Culture of *C. difficile* and preparation of purified SLPs

*C. difficile* (PCR Ribotype 001; toxin A and B positive; clindamycin resistant; HPA UK reference R13537, Anaerobe Reference Unit, Public Health Laboratory, University Hospital of Wales) isolated from a patient with *C. difficile*-associated disease was used for preparation of SLPs as previously described [6]. Briefly, SLPs were purified from cultures grown anaerobically at 37°C in BHI/0.05% thioglycolate broth. Cultures were harvested and crude SLP extracts dialysed and applied to an anion exchange column attached to an AKTA FPLC system (MonoQ HR 10/10 column, GE Healthcare).

The pure SLPs were eluted with a linear gradient of 0–0.3 mol/L NaCl at a flow rate of 4 mL/min. Peak fractions corresponding to pure SLPs were analysed on 12% SDS–PAGE gels stained with Coomassie blue and assessed for LPS contamination using a *Limulus* amoebocyte lysate (LAL) assay. The individual SLPs (high and low molecular weight) were separated by chromatography under the same conditions, but with 8 M urea included in all buffers. The urea was then dialysed out. Additional fractions containing irrelevant proteins were also kept for comparison.

### Isolation and culture of bone marrow-derived DCs

Bone marrow-derived immature DCs (BMDCs) were prepared by culturing bone marrow cells obtained from the femurs and tibia of mice in RPMI 1640 medium with 10% fetal calf serum (cRPMI) supplemented with 10% supernatant from a GM-CSF-expressing cell line (J558-GM-CSF). The cells were cultured at 37°C for 3 days, and the supernatant was carefully removed and replaced with fresh medium with 10% GM-CSF cell supernatant. On day 7 of culture, cells were collected, counted, and plated at 1×10^6^/mL for experiments.

### Dendritic cell: T cell co-culture

BMDCs from C3H/HeN and C3H/HeJ mice were cultured and activated with ovalbumin (OVA) peptide (323–339; 5 µg/mL) in the presence of either LPS (100 ng/mL) or SLPs (20 µg/mL) for 24 h. After 24 h, DCs were collected and washed twice in sterile PBS/2% FCS and irradiated with 40 Gy (4000 rads) using a gamma irradiator with a Caesium-137 source. A final concentration of 2×10^5^cells/mL were added to CD4^+^ T cells, isolated from the spleens of OVA transgenic D011.10 mice (2×10^6^ cells/mL) and incubated. On day 5 of co-culture, the supernatant was removed and frozen for cytokine analysis. Fresh medium was added, and the cells were incubated until day 7 and supernatants removed. Newly harvested OVA/SLP or OVA/LPS-activated DCs were added (2×10^5^ cells/mL) with recombinant murine IL-2 (10 U/mL; Becton Dickinson) for the second round of T cell stimulation. At the end-point of the experiment (day 10), supernatants were removed and frozen for cytokine analysis.

### Enzyme-linked immunosorbent assay (ELISA)

DCs were incubated with either SLP (20 µg/mL) or LPS (100 ng/mL) for 24 h. Culture supernatants from this experiment as well as the DC:T cell co-culture experiments were removed and stored at −80°C until analysis. TNF-α, IL-1β, IL-10 and IL-12p70, IL-12p40, IL-23, IFNγ, IL-17 and IL-4 concentrations in cell culture supernatants were analysed by DuoSet ELISA kits (R&D Systems), according to the manufacturer's instructions.

### Flow cytometry

DCs were cultured as previously described and incubated with either SLPs (20 µg/mL) or LPS (100 ng/mL) for 24 h. In some experiments a p38 inhibitor (S8308; 20 µg/mL) was used. Cells were then washed and used for immunofluorescence analysis. The expression of CD40, CD80, CD86 and MHCII was assessed using an anti-mouse CD11c (Caltag), and CD40, CD80, CD86 and MHCII (rat IgG2a, BD Biosciences) and appropriately labelled isotype-matched antibodies. After incubation for 30 min at 4°C, cells were washed and immunofluorescence analysis was performed on a FACsCalibur (BD Biosciences) using Cell Quest software.

### ISRE and NF-κB luciferase assays

Human HEK293-TLR4, HEK293-MD2-CD14-TLR4 and HEK293T were transiently transfected using GeneJuice transfection reagent (Novagen, Madison, WI) according to the manufacturer's instructions with a total amount of 220 ng DNA per well comprising of 75 ng ISRE- (Clontech, Palo Alto, CA) or κB-luciferase plasmid, 30 ng *Renilla*-luciferase and empty pcDNA3.1 vector as filler DNA. 24 h after transfection, cells were stimulated with LPS (100 ng/mL) or SLPs (0–100 µg/mL) for 6 h before lysis. Firefly luciferase activity was assayed by the addition of 40 µl of luciferase assay mix to 20 µl of the lysed sample. *Renilla*-luciferase was read by the addition of 40 µl of a 1∶1000 dilution of Coelentrazine (Argus Fine Chemicals) in PBS. Luminescence was read using the Reporter microplate luminometer (Turner Designs). The *Renilla*- luciferase plasmid was used to normalise for transfection efficiency in all experiments.

### Preparation of *C. difficile* inoculum

*C. difficile* (R13537), described above, was grown on blood agar plates under anaerobic conditions at 37°C for 5 days to generate spores. Spore inoculum was prepared as described by Sambol *et al.* [24], the spore concentration was determined by dilution plating onto blood agar plates and stock solutions of 5×10^6^ spores ml^−1^ were stored at −80°C.

### *C. difficile* infection of mice

TLR2^−/−^, TLR4^−/−^, MyD88^−/−^, TRIF^−/−^ and wild-type mice, all on a C57BL/6J strain background, were infected with *C. difficile* using an antibiotic-induced model of mouse infection [25]. Mice were treated for 3 days with an antibiotic mixture of kanamycin (400 µg/ml), gentamicin (35 µg/ml), colistin (850 U/ml), metronidazole (215 µg/ml) and vancomycin (45 µg/ml) in the drinking water. Mice were subsequently given autoclaved water. On day 5, mice were injected i.p. with clindamycin (10 mg/kg). Mice were infected with 10^3^ *C. difficile* spores on day 6 by oral gavage. Initial studies determined infection with 10^3^ spores of *C. difficile* R13537 caused mild transient weight loss and diarrhoea in wild-type C57BL/6J strain mice. Mice that were not treated with antibiotics were also challenged with *C. difficile*. Animals were weighed daily and monitored for overt disease, including diarrhoea. Moribund animals with >15% loss in body weight were humanely killed. The cecum was harvested from uninfected (day 0) and infected mice at days 3 and 7 and the contents were removed for CFU counts. The cecum was fixed in 10% formaldehyde saline and paraffin sections were hematoxylin and eosin-stained. Evaluation of histopathology was performed as previously described [26]. Briefly slides were scored by two independent investigators, blinded to the study groups, on a 0–3 scale as follows; absence of inflammation and damage was scored 0, while mild, moderate and severe inflammatory changes were scored 1, 2 and 3 respectively. The severity of mucosal damage and inflammation was based on the levels of mucosal epithelial damage and erosion, cell inflammation of the lamina propria, crypt abscess formation as well as the incidence and severity of oedema.

### CFU counts

The contents of cecum were recovered from infected and uninfected mice, weighed and stored frozen. Each sample of cecum material was thawed and homogenised in 1 ml PBS (pH 7.4) by vortex mixing in a 1.5 ml microcentrifuge tube. The suspension was serially diluted (10^−1^ to 10^−4^) and 50 µl of each dilution was spread in duplicate onto quadrants of Brazier's CCEY plates (Lab M). Plates were incubated under anaerobic conditions at 37°C for 30 h. Colonies were counted and CFU/g determined for each sample.

### Measurement of antibody response to SLP by ELISA

The anti-SLP IgG was measured as previously described [5]. Briefly plates were coated overnight with 2 µg/mL and blocked for 1 h with blocking buffer (PBS containing 2% nonfat dry milk). Serum samples were diluted 1∶50 and further serial 10-fold dilutions of samples were made in antibody buffer (blocking buffer containing 0.05% Tween 20). Bound antibody was detected with HRP-conjugated anti-mouse IgG followed by TMB. Reactions were stopped with 1 M H_2_SO_4_, and ODs were read at 450 nm.

### Statistical analysis

One-way analysis of variance (ANOVA) was used to determine significant differences between conditions. When this indicated significance (p<0.05), post-hoc Student-Newmann-Keul test analysis was used to determine which conditions were significantly different from each other.

## Results

### Characterisation of SLPs from *C. difficile*

**Figure 1:**
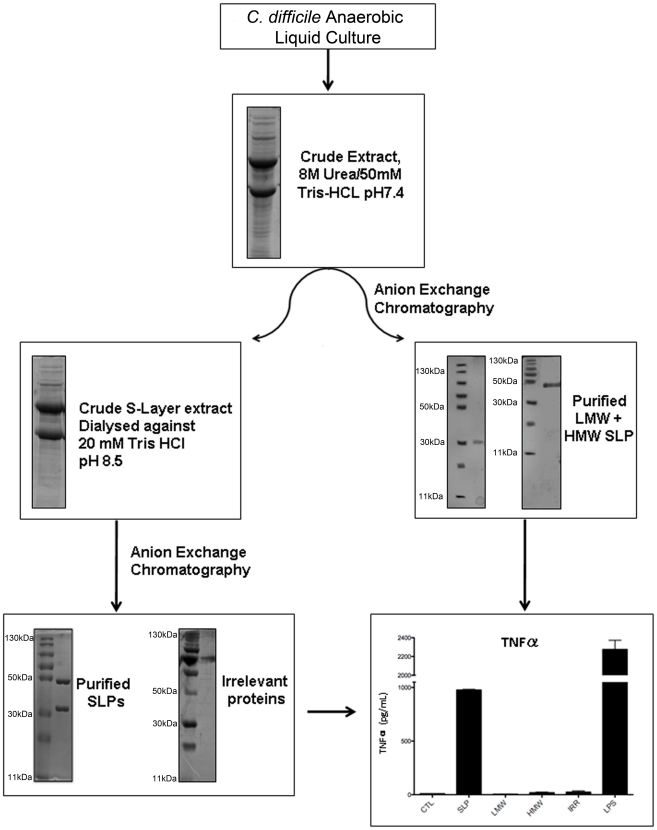
Purification and characterisation of SLPs isolated from *C. difficile*. Coomassie blue stained 12% SDS-PAGE gel showing crude extracts and purified *C. difficile* SLPs at each stage of the purification process. Individual high molecular weight (HMW) and low molecular weight (LMW) proteins and an irrelevant protein from the same process are also shown. Bone marrow derived DCs were isolated from Balb/C mice and incubated with either SLPs (20 µg/mL), LMW (20 µg/mL), HMW (20 µg/mL) or an irrelevant protein (20 µg/mL) for 24 h. LPS (100 ng/ml) was used as a positive control. The concentration of TNFα was measured in the supernatants by ELISA. The results are the mean (±SEM) for n = 4. *** p<0.001, determined by one-way ANOVA test comparing all groups.

Samples from all stages of the purification process were run on SDS-PAGE gels to demonstrate the purity of the SLPs. Figure 1 clearly shows the presence of multiple bands in the crude extract and only two bands with molecular masses of 42–48 kDa and 32–38 kDa following anion exchange chromatography. Furthermore, we also purified individual high molecular weight (HMW) and low molecular weight (LMW) proteins which were also seen as single bands at the correct molecular weight on SDS gels. In order to confirm that any activity by the SLPs was attributed to the protein and not a contaminant, we also examined irrelevant proteins which were purified in the same manner but were eluted in different fractions to those of the SLPs.

In order to assess whether SLPs could activate DCs we examined their ability to induce TNFα production by these cells. The graph in Figure 1 shows that SLPs induce TNFα production by DCs. This is not seen with the individual LMW and HMW proteins or the irrelevant protein. LPS was used as a positive control. The ability of SLPs to induce cytokine secretion in DCs was found to be dose dependent (Figure S2).

### SLPs from *C. difficile* induce cytokine production in BMDC via a TLR4-dependent mechanism

**Figure 2:**
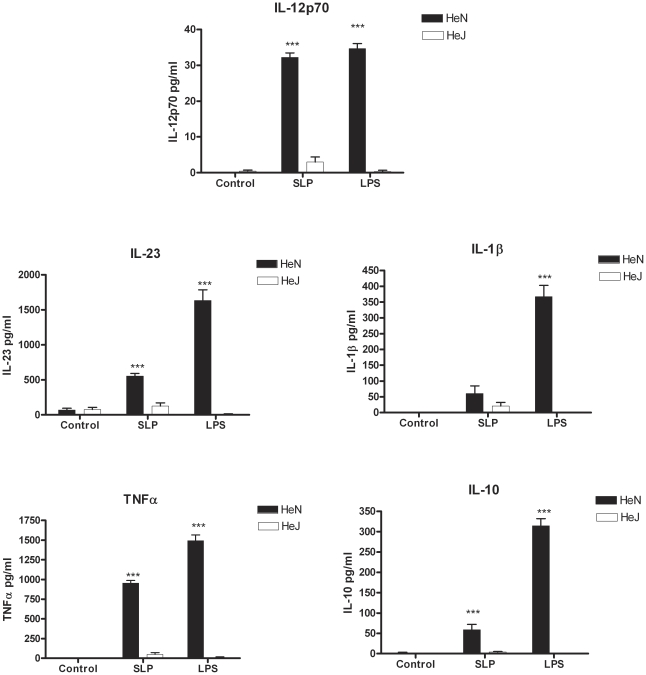
SLPs induce cytokine production in BMDC via TLR4. DCs isolated from C3H/HeN or C3H/HeJ mice were incubated with LPS (100 ng/mL) or SLPs (20 µg/mL) for 24 h. Concentrations of IL-12p70, IL-23, IL-1β, TNFα and IL-10 were measured in the supernatants by ELISA. The results are the mean (±SEM) for n = 4. *** p<0.001, determined by one-way ANOVA test comparing all groups.

As SLPs and LPS induced DCs to produce a similar profile of cytokines, we examined whether SLPs also activated DCs via TLR4. Given that the differentiation of naïve CD4^+^ T cells into Th subsets is determined in part by the cytokines produced by DCs upon activation [17], we specifically examined the effects of SLPs on these cytokines. Incubation of BMDCs isolated from C3H/HeN with SLPs induced significant production of IL-12p70 (Figure 2; *p*<0.001), IL-23 (Figure 2; *p*<0.001) and IL-10, important for Th1, Th17 and Tr1 responses respectively, and also significant levels of TNFα (Figure 2; *p*<0.001). Interestingly, there was no significant induction of IL-1β by SLPs. The effects of both LPS and SLPs on cytokine production were completely absent in BMDCs from C3H/HeJ mice, indicating that the activation of DCs by SLPs occurs via TLR4.

### SLPs from *C. difficile* induce DC maturation via a TLR4-dependent mechanism

**Figure 3:**
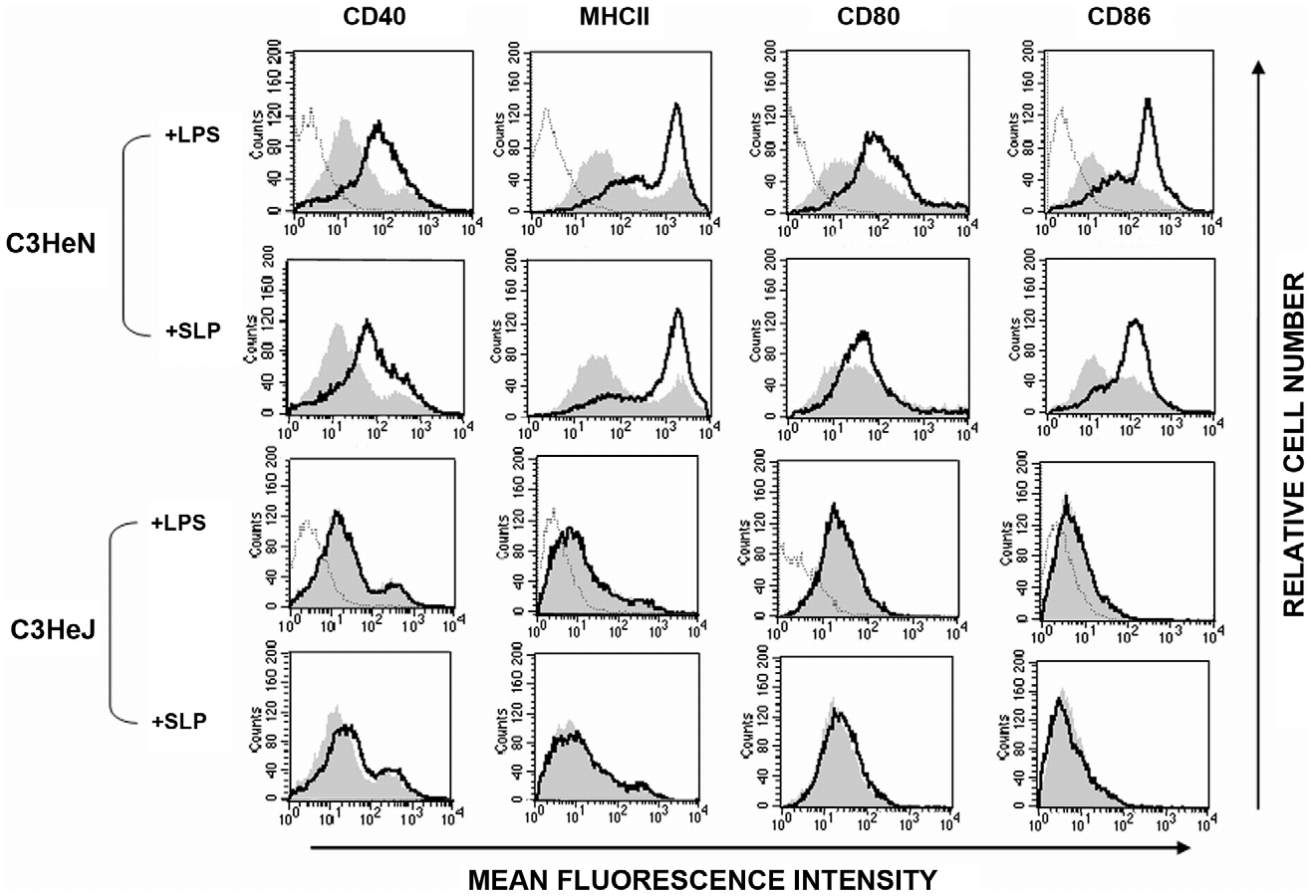
SLPs induce DC maturation via TLR4. DCs isolated from C3H/HeN or C3H/HeJ mice were incubated with either LPS (100 ng/mL) or SLPs (20 µg/mL) for 24 h. Cells were washed and stained with antibodies specific for CD40, CD80, CD86, and MHCII, or with isotype matched controls. Results of immunofluorescence analysis are shown for control DCs (filled histogram) and DCs treated with either LPS or SLPs (thin black line). Histograms for isotype controls are shown as dotted lines in the first row only.

**Figure 4:**
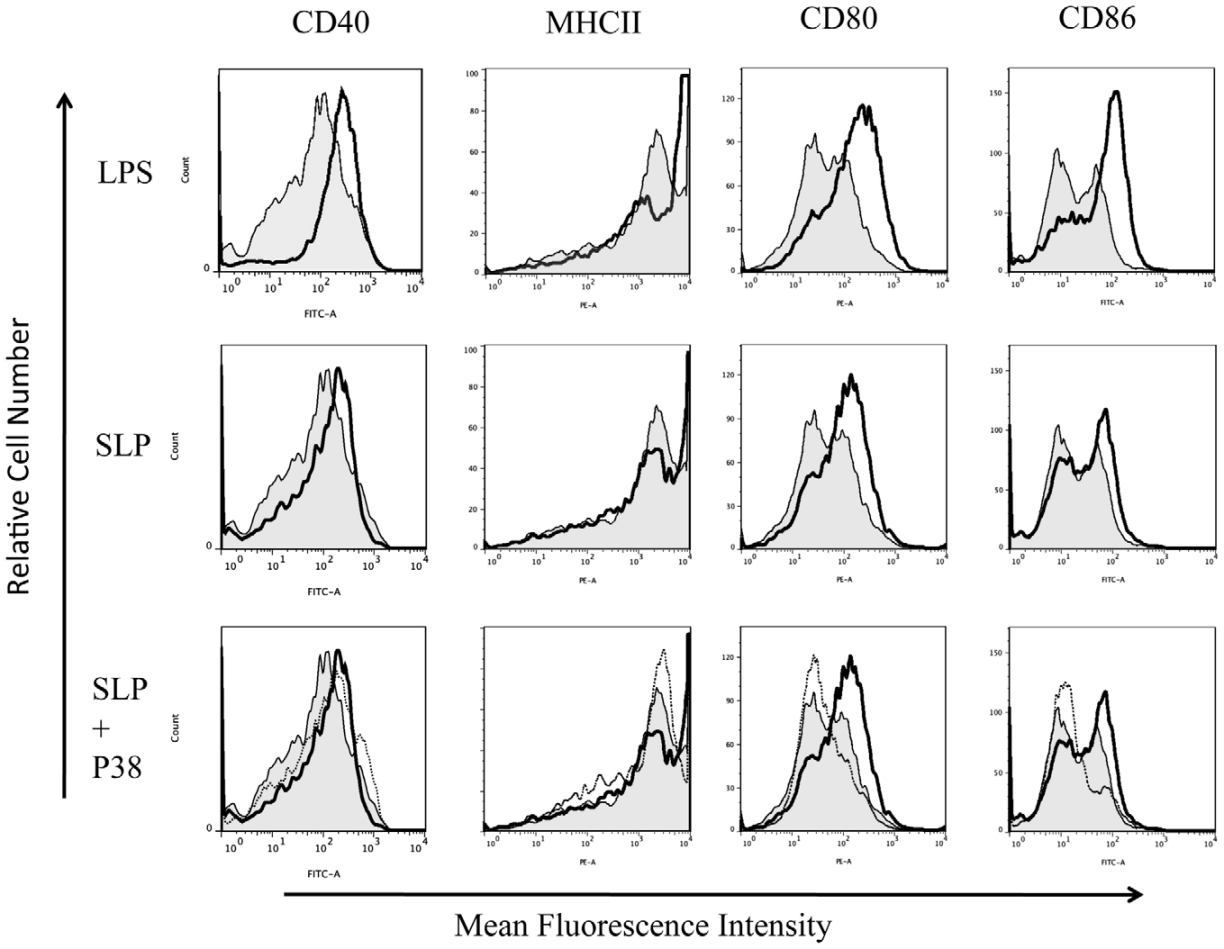
SLP-induced DC maturation is dependent on p38. DCs isolated from Balb/C mice were incubated with either LPS (100 ng/mL), SLPs (20 µg/mL) or SLPs plus the p38 inhibitor S8308 (20 µg/mL) S for 24 h. Cells were washed and stained with antibodies specific for CD40, CD80, CD86, and MHCII, or with isotype matched controls. Results of immunofluorescence analysis are shown for control DCs (filled histogram) and DCs treated with either LPS or SLPs (thin black line) in the rows one and two. In row three, control DCs (filled histogram) are represented along with SLP (dark black line) and SLPs plus S8308 (thin black line).

DC maturation is also characterized by increased expression of MHC class II, CD40, CD80 and CD86 [27], [28]. As SLPs activated DCs via TLR4, we examined the effects of SLPs on these markers in the presence and absence of TLR4. Figure 3 demonstrates that SLPs induce DC maturation in a similar manner to LPS, in cells isolated from C3H/HeN mice with increased expression of MHC II, CD40, CD80 and CD86. This was completely abrogated in DCs isolated from C3H/HeJ TLR4 mutant mice. Activation of TLR4 results in the subsequent phosphorylation and activation of p38. In order to further confirm that SLP activated DCs via TLR4 we examined the ability of SLP to induce DC maturation in the presence of a p38 inhibitor. Figure 4 demonstrates that SLP is unable to induce upregulation of MHC II, CD40, CD80 or CD86 in the presence of a p38 inhibitor. Furthermore, the effects of SLPs on DC maturation markers was also dose dependent (Figure S3).

### SLPs from *C. difficile* fail to induce adaptive immunity in the absence of TLR4

**Figure 5:**
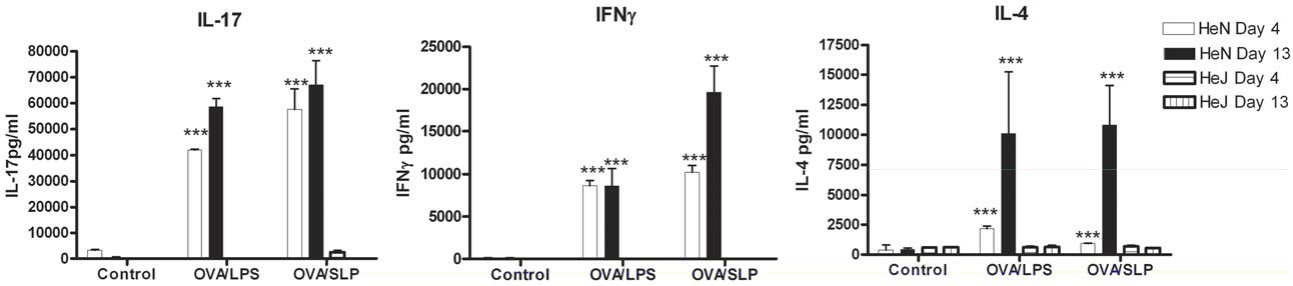
DCs activated by SLPs induce T helper responses. DCs isolated from C3H/CheN or C3H/CheJ mice were incubated with LPS (100 ng/mL) or SLPs (20 µg/mL) in the presence of OVA peptide (5 µg/mL) for 24 h. After 24 h DC (2×10^5^/mL) were irradiated and added to CD4^+^ T cells (2×10^6^/mL) purified from the spleens of OVA D011.10 mice. Supernatants were removed on Day 4. Fresh SLP/OVA- or LPS/OVA-treated DC were added on Day 7, together with rIL-2 (10 U/mL). Supernatants were removed on Day 10, and analysed for IFNγ, IL-17, and IL-4 using ELISA. Results are means (± SEM) of n = 3 and represent two independent experiments. *** *p*<0.001, determined by one-way ANOVA test comparing all groups.

An important event for the initiation of adaptive immunity is the activation of Th cells by DCs [29]. The DC cytokine production and co-stimulatory marker expression are key to this process. We first wanted to determine whether SLPs could induce a Th1 or Th17 response, given the importance of these responses in bacterial clearance [30], [31]. Furthermore, since our earlier data demonstrated that activation of DCs by SLPs involves TLR4, we wanted to determine whether this was critical for generation of subsequent adaptive immune responses. DCs isolated from both C3H/HeN and C3H/HeJ mice were exposed to OVA peptide in the presence of either SLPs or LPS. These DCs were then co-cultured with CD4^+^ T cells purified from OVA transgenic mice. T cells were exposed to two rounds of activation with DCs and the Th response was characterised. DCs activated with LPS/OVA or SLP/OVA induced a mixed T helper cell response, with significant production of IL-17, IL-4 and IFNγ on both Day 4 and Day 10 (Figure 5; *p*<0.001). The dominant response was the production of IL-17. No response was generated by either LPS/OVA- or SLP/OVA-activated DCs isolated from C3H/HeJ mice.

### SLPs activate NF-κB downstream of TLR4 activation in human HEK293 cells independently of CD14

**Figure 6:**
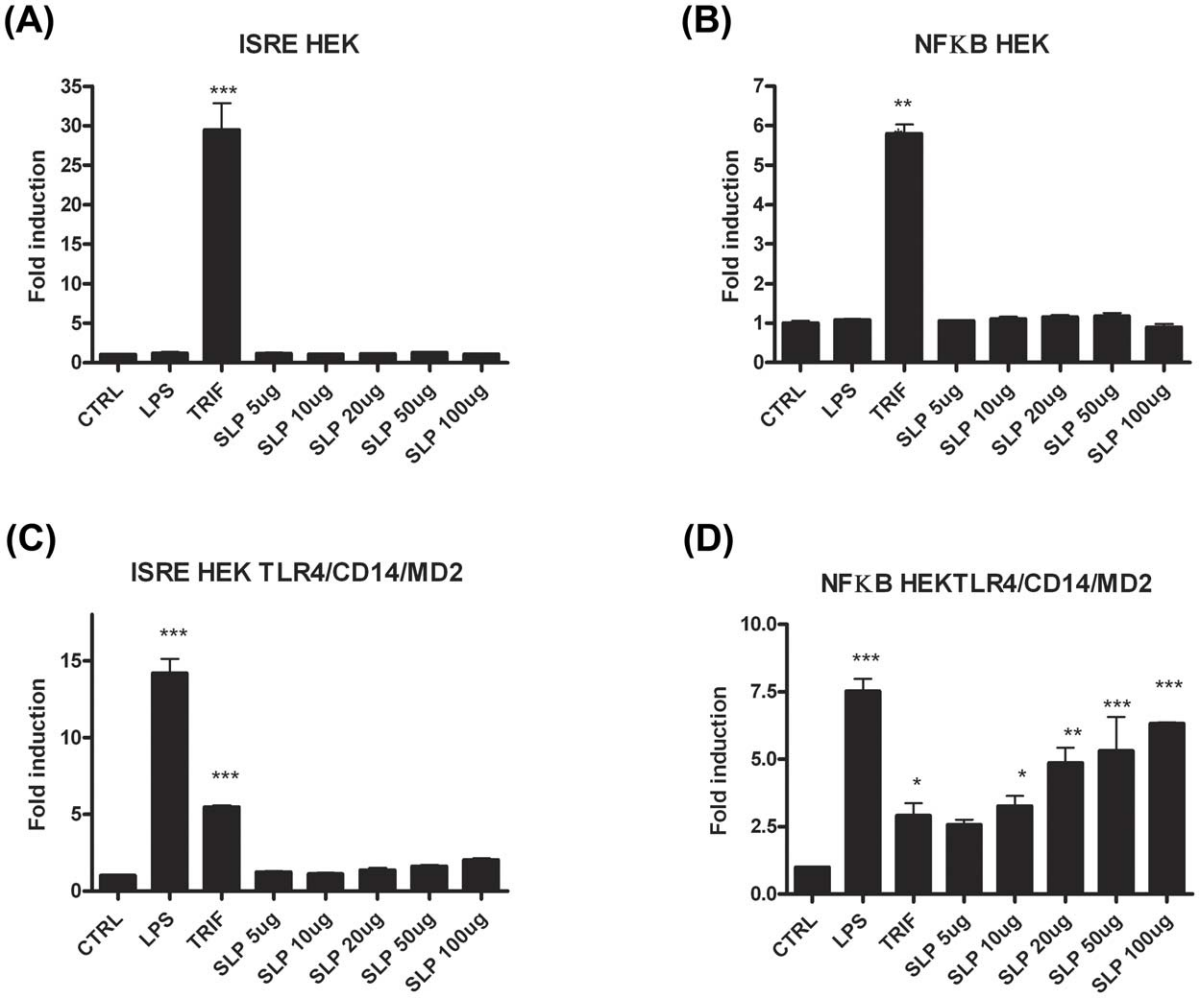
SLPs activate NFκB downstream of TLR4 but not IRF3. HEK293T cells and HEK293-MD2-CD14-TLR4 cells were transiently transfected with ISRE-luciferase (75 ng) and *Renilla*-luciferase (30 ng). 24 h after transfection, the cells were stimulated with LPS (100 ng/mL) or SLP (5–100 µg/mL) for 6 h. The cells were lysed, and luciferase activity as an indicator of ISRE activity (A&C) or NFκB activity (B&D) was subsequently measured. Results were normalised for *Renilla*-luciferase activity and represented as fold stimulation over the non-stimulated control. Results are expressed as mean (± SEM) for duplicate determinations and are representative of two separate experiments. *** *p*<0.001, determined by one-way ANOVA test.

In order to confirm that SLPs activate TLR4, we performed experiments in which human HEK293 cells were transiently transfected with TLR4 along with the TLR4 accessory proteins, MD2 and CD14. Non-transfected HEK293 cells were used as a control. Two separate experiments were carried out using luciferase as a reporter gene for activation of the transcription factors NFκB or ISRE (indicative of interferon regulatory factor 3 (IRF3) activation). As expected, neither LPS nor SLP were able to activate ISRE or NFκB in HEK293 cells in the absence of the TLR4 receptor (Figure 6A&B). Exposure of HEK293-TLR4-MD2-CD14 cells to LPS resulted in significant activation of ISRE and NFκB (Figure 6C&D; *p*<0.001). When increasing concentrations of SLPs were incubated with the HEK293-TLR4-MD2-CD14 cells, there was a dose-dependent activation of NFκB (Figure 6D; *p*<0.05, *p*<0.01, *p*<0.001), but no activation of ISRE (Figure 6C).

**Figure 7:**
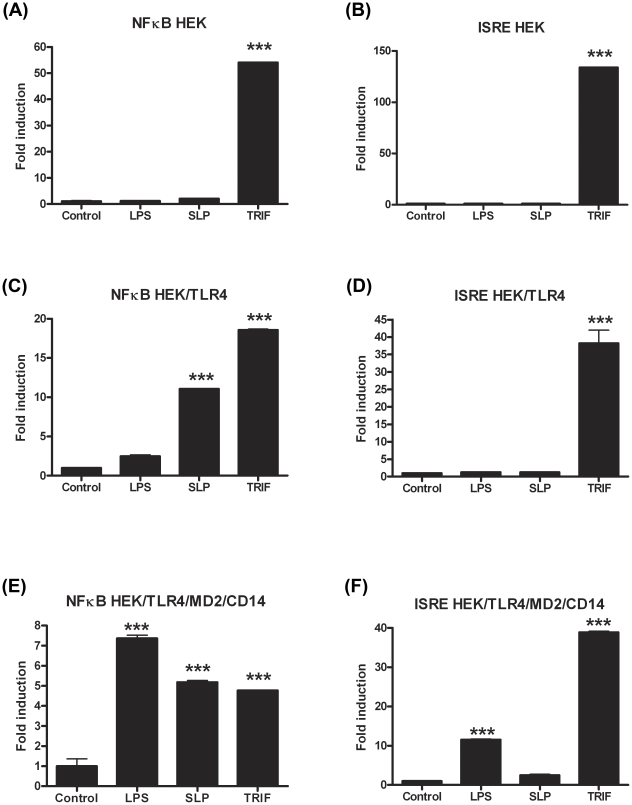
SLPs activate NFκB independently of CD14. HEK293T cells, HEK293T-TLR4 cells and HEK293-MD2-CD14-TLR4 cells were transiently transfected with ISRE-luciferase (75 ng) and *Renilla*-luciferase (30 ng). Twenty-four hours after transfection, the cells were stimulated with LPS (100 ng/mL) or SLP (20 µg/mL) for 6 h. The cells were lysed, and luciferase activity as an indicator of ISRE activity (B, D, F) or NFκB activity (A, C, E) was subsequently measured. Results were normalised for *Renilla*-luciferase activity and represented as fold stimulation over the non-stimulated control. Results are expressed as mean (± SEM) for duplicate determinations and are representative of two separate experiments. *** *p*<0.001, determined by one-way ANOVA test.

The lack of effect of SLPs on IRF3 was further confirmed by our observation that SLP did not induce IFNβ production by DCs (Figure S4). Given that SLP did not activate IRF3, and that CD14 is important for the endocytosis of the TLR4 complex for subsequent activation of IRF3 [32], we examined whether SLP required CD14 for activation of TLR4. We show that LPS activated NF-κB in HEK293-TLR4-MD2-CD14 (Figure 7C) cells but not HEK293-TLR4 cells (Figure 7E). In contrast, SLP significantly induced NF-κB in both HEK293-TLR4 (Figure 7E) and HEK293-TLR4-MD2-CD14 cells (Figure 7C; *p*<0.001), suggesting that SLP does not require CD14 for activation of NF-κB downstream of TLR4.

### TLR4^−/−^ and MyD88^−/−^ mice are more susceptible to *C. difficile* infection

**Figure 8:**
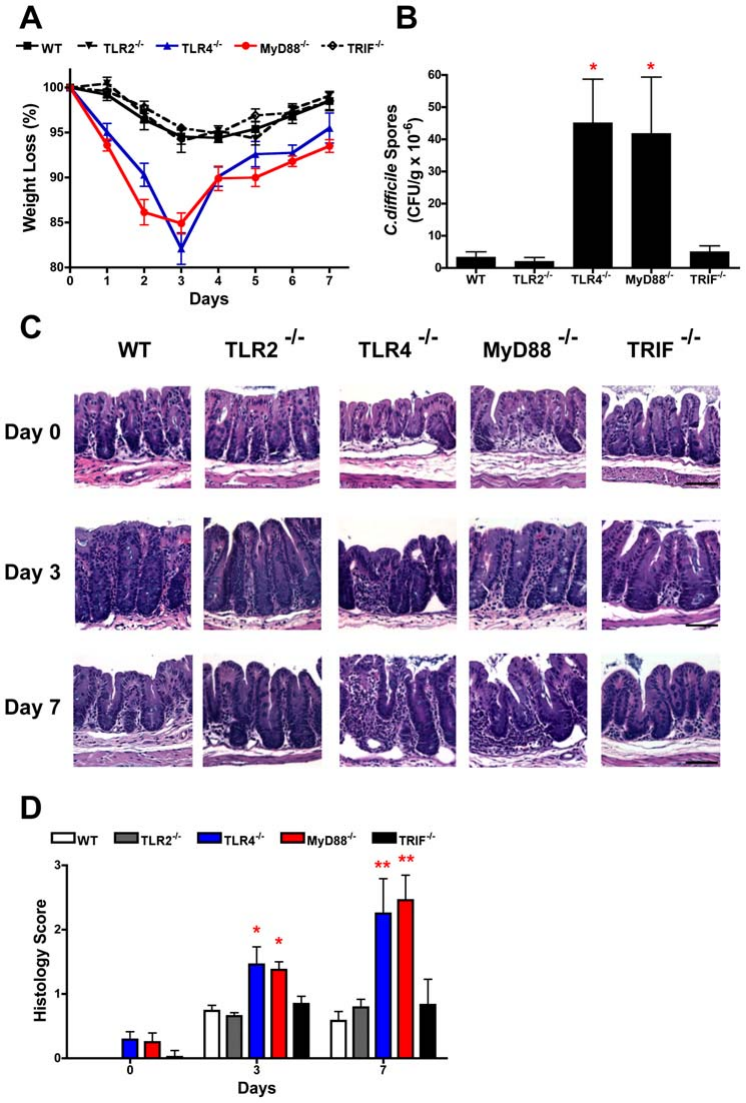
TLR4^−/−^ and MyD88^−/−^ mice show increased severity of infection with *C. difficile* infection. Mice were pre-treated with antibiotics and infected with *C. difficile* (see materials and methods). (A) Change in body weight of groups of mice following infection; 6–8 mice per group, data are expressed as mean ± SEM. (B) CFUs in the cecum on day 3 of infection; 3–4 mice per group, data are expressed as mean ± SEM. (C) Representative hematoxylin and eosin-stained sections of the cecum from uninfected (day 0) mice and from animals 3 and 7 post-infected. Original magnification ×200, scale bar 50 µM. (D) Mean histology scores (range 0–3) of sections of the cecum before (day 0) and 3 and 7 days post-infection. Statistical differences between groups was determined by ANOVA, * *p*<0.05; ** *p*<0.01, *** *p*<0.001.

To formally validate the biological relevance of the *in vitro* cell culture data indicating that *C. difficile* SLPs interact with TLR4, wild-type, TLR2^−/−^, TLR4^−/−^, MyD88^−/−^ and TRIF^−/−^ mice were infected with the *C. difficile* strain that SLPs were isolated from. A recently described model of *C. difficile* infection of antibiotic treated mice was used [25]. Following infection wild-type, TLR2^−/−^ and TRIF^−/−^ mice developed comparable diarrhoea and transient weight loss, which peaked at day 3 post-infection (Figure 8A). In contrast, both TLR4^−/−^ and MyD88^−/−^ mice developed marked weight loss by day 1, with significantly greater weight loss (*p*<0.05−0.001) relative to other groups on days 1–7 (Figure 8A), which was associated with severe diarrhoea. Due to severe morbidity and associated >15% weight loss, 1/7 and 2/7 of TLR4^−/−^ and MyD88^−/−^ groups were humanely killed on day 3, respectively, with no deaths in wild-type, TLR2^−/−^ or TRIF^−/−^ mice. Consistent with the weigh loss data, both TLR4^−/−^ and MyD88^−/−^ mice had significantly (*p*<0.05) higher numbers of *C. difficile* spores in the cecum on day 3 compared to wild-type, TLR2^−/−^ and TRIF^−/−^ (Figure 8B). The cecum from TLR4^−/−^ and MyD88^−/−^ had marked inflammatory cell infiltrates with oedema and epithelial disruption on days 3 and 7 post infection, that was significantly (*p*<0.05−0.01) greater than the mild inflammation in the cecum of infected wild-type, TLR2^−/−^ or TRIF^−/−^ mice (Figure 8C, 8D). It was notable that uninfected TLR4^−/−^ and MyD88^−/−^ mice had evidence of mild cecal inflammation (Figure 8C, 8D), which is relevant to the known role of TLR4 and MyD88 in basal intestinal homeostasis [33], [34]. As conventional housed mice are not susceptible to *C. difficile* infection, we evaluated if the intestinal alterations in TLR4^−/−^ and MyD88^−/−^ mice rendered these mice innately more susceptible to infection. However, TLR4^−/−^ and MyD88^−/−^ mice, and also wild-type, TLR2^−/−^ and TRIF^−/−^ mice, were refractory to infection when exposed to *C. difficile* without any prior antibiotic treatment (data not shown). These data confirm an *in vivo* functional role for TLR4, and not TLR2, in a MyD88 but not TRIF dependent pathway, in *C. difficile* infection of antibiotic-treated mice.

### TLR4^−/−^ mice show no IgG response to SLP following infection with *C. difficile*

**Figure 9:**
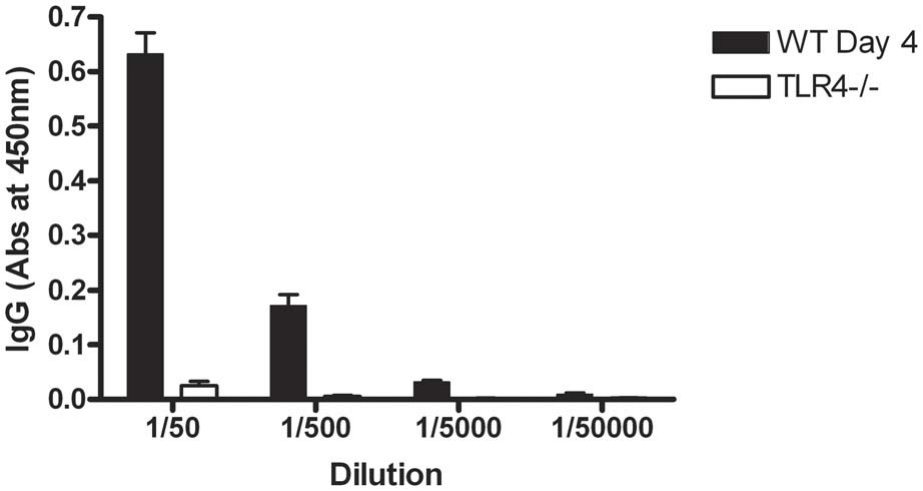
TLR4^−/−^ mice do not develop an antibody response to SLPs following infection. Mice were pre-treated with antibiotics and infected with *C. difficile* (see materials and methods). Serum was collected on day 3 of infection and the levels of anti-SLP IgG were measured using ELISA. Results are shown as absorbance values for wildtype versus TLR4−/− mice on day 3 of infection at a range of dilutions of serum. Results are expressed as mean ± SEM.

In order to confirm that SLPs were recognised in the context of the whole bacterium and that TLR4 is necessary for their recognition, wildtype and TLR4^−/−^ mice were infected as before with *C. difficile* and serum was collected 3 days post infection. Wildtype mice showed an increase in anti-SLP IgG 3 days after infection with *C. difficile* (data not shown). Figure 9 demonstrates that TLR4^−/−^ mice have no detectable anti-SLP IgG compared to wildtype controls on day 3 post infection.

## Discussion

The significant findings of this study are that SLPs isolated from *C. difficile* induce maturation of DCs and subsequent generation of T helper cell responses required for bacterial clearance via TLR4. We also demonstrate the significance of TLR4 in murine infection with *C. difficile*, with TLR4^−/−^ and MyD88^−/−^ mice displaying a more severe infection than wild type. Interestingly, we found SLPs to activate NFκB but not IRF3, downstream of TLR4 which correlated with the observation that TRIF^−/−^ mice did not have increased susceptibility or severity of infection. This is the first study to demonstrate a role for TLR4 in infection associated with *C. difficile* and suggests an important role for SLPs in the generation of the immune response necessary for clearance of this bacterium.

SLPs have previously been described as virulence factors for other bacterial infections such as *Campylobacter fetus* and *Aeromonas salmonicida* [8], [9]. There is now significant evidence that SLPs isolated from *C. difficile* are important components of the pathogen. Specifically, passive immunisation of hamsters with antibodies to these proteins affects the course of *C. difficile* infection, resulting in prolonged survival of hamsters [6]. While this evidence indicates the importance of these proteins, the way in which they are recognised by and activate the immune system is not clear.

Activation of DCs is characterized by the production of cytokines and increased expression of MHCII, as well as co-stimulatory molecules [27], [29]. We demonstrate that SLPs induce DC maturation, characterised by production of IL-12p70, TNFα, IL-23, IL-6, and increased expression of MHCII, CD40, CD80 and CD86. This agrees with some previously reported effects of SLPs [35]. Interestingly, while there are some similarities between the response elicited with SLP and LPS, SLP did not induce IL-1β production, demonstrating a distinct effect of SLPs and further confirming that potential contamination with LPS is not responsible for the effects observed with SLPs. Other evidence was provided by our observation that the effects of SLPs on DCs were not reversed in the presence of polymyxin B, known to bind LPS (Figure S1).

We next conducted experiments in C3H/HeN and C3H/HeJ mice, and showed that the effects of SLPs on DC maturation were mediated through TLR4. Furthermore, our experiments demonstrated that intact SLPs, containing both the HMW and LMW proteins, were required for DC activation. The significance of this data is two-fold; firstly they demonstrate that the HMW and LMW proteins may need to be associated in their complex for recognition by TLR4; while an additional experiment examining the HMW and LMW proteins after recomplexing would be advantageous, their tight association has been recently demonstrated by Fagan *et al.* [36]. Secondly, the lack of response to the separated proteins confirms that the effects we observed with SLPs could not possibly be attributed to any contaminating ligand. This is further supported by the fact that an irrelevant protein purified in the same way was unable to elicit these effects on DCs.

A number of pathogen derived molecules have now been shown to activate DCs through TLR4. For example, LPS from *Bordetella pertussis* and *Salmonella enteritidis* have been reported to induce TLR4-dependent DC maturation [27], [37]. Given that the interaction of DC with T cells is required for activation of adaptive immunity, and since SLPs induced potent production of cytokines important in promoting Th1 and Th17 responses [17], [18], the data suggest that SLPs may be important in the generation of these responses.

We clearly demonstrate that DCs activated with SLPs have the capacity to drive strong Th1 and Th17 responses characterised by production of IFNγ and IL-17. Indeed the dominant cytokine produced was IL-17. Not surprisingly, SLPs also induced a weak Th2 response, which concurs with studies demonstrating SLPs to induce an antibody response [6], [7]. The importance of T helper cells and their cytokines IFNγ and IL-17 are well recognised in bacterial clearance. Both *Scid* mice (deficient in T and B cells) and nude mice (deficient in T cells) show high susceptibility to infection with *Coxiella burnetii* [19]. Another study employing IFNγ^−/−^ mice demonstrated that infection with *Bordetella pertussis* was exacerbated in the absence of IFNγ [38]. Furthermore, inhibition of IL-17 with a neutralising antibody results in increased infection with *Pneumocystis carinii* [39]. Since SLPs induce a potent Th1 and Th17 response, our data suggest that they may be important in clearance of *C. difficile* and that TLR4 is required for this.

Several studies have highlighted a role for TLR4 in bacterial clearance; for example activation of TLR4 by *Klebsiella pneumoniae* has been shown to be critical for induction of IL-17, known to be important in host defence against bacterial infection [40]. Therefore, we examined whether clearance of *C. difficile* was impaired in mice without functional TLR4. We clearly demonstrate that *C. difficile* infection is more severe in TLR4^−/−^ and MyD88^−/−^ mice with increased weight loss, mortalities, and number of *C. difficile* spores in the cecum. This suggests that TLR4 and MyD88-mediated signalling are important in the clearance of the bacterium. Furthermore, the lack of an IgG response to SLP in TLR4^−/−^ mice suggests that the recognition of SLP plays a key role in this process. Our findings in MyD88^−/−^ mice concurs with a recent study which showed a more severe intestinal disease following infection with *C. difficile* in these mice [41]. It is noteworthy that while TLR4^−/−^ and MyD88^−/−^ mice were relatively more susceptible to infection following an antibiotic treatment regime, however, without antibiotic treatment and thus having an intact intestinal microbiota they were resistant to *C. difficile* infection infected similar to immunocompetent C57BL/6J mice. Recently, Jordan *et al* demonstrated that mice deficient in functional TLR4 showed increased susceptibility to infection with *Rickettsia conorii* which was associated with decreased Th1 and Th17 responses [42]. Importantly, rickettsiae do not possess classical endotoxic LPS. Given that *C. difficile* is a Gram-positive bacterium lacking LPS, our findings that SLPs, the immunodominant antigen on the surface of this bacterium, can activate the innate immune response via TLR4 are particularly significant.

Recognition and subsequent binding of LPS to TLR4 results in an intracellular cascade of events involving the adaptor molecules MyD88, MyD88-like adaptor molecule (Mal), TRIF and TRIF-related adaptor molecule (TRAM), culminating in downstream activation of the transcription factors NFκB and IRF3 for production of pro-inflammatory cytokines and type I interferons, respectively [43], [44]. In order to confirm that SLPs can indeed activate TLR4, we examined whether they induced activation of NFκB and IRF3 in human HEK cells transfected with TLR4-MD2-CD14. We demonstrate that SLPs activate NFκB in a dose dependent manner downstream of TLR4, however they did not activate ISRE which is indicative of IRF3 activation. The significance of this data is two-fold; firstly, it raised the possibility that SLPs activated TLR4 independently of CD14; given that activation of IRF3 downstream of TLR4 requires endocytosis of the TLR4 complex and its subsequent association with TRIF and TRAM [15]; this finding explains why our experiments in HEK/TLR4 cells clearly show that SLP, but not LPS, activated NKκB in the absence of CD14. This is further supported by our data showing that mice deficient in TRIF did not get a more severe infection and our data showing that SLP does not induce type 1 IFN in DCs (Figure S4). Secondly, these data further confirm that our purified SLPs were free from LPS contamination as LPS clearly activates ISRE. A recent report has highlighted the ability of some TLR4 ligands to selectively activate signalling pathways downstream of TLR4. Specifically, the vaccine adjuvant monophosphoryl lipid A induces strong TRIF-associated responses but only very weak MyD88-associated responses, showing a clear preference for activation of downstream IRF3 [45]. Interestingly, as production of IFNβ (downstream of IRF3 activation) is essential for induction of endotoxic shock, the inability of SLPs to activate the ISRE/IRF3 pathway and subsequent IFNβ may explain why numerous groups that administer SLPs to mice do not report any toxicity [46], [47].

The data presented in this study demonstrate that SLPs activate innate and adaptive immunity via a TLR4-dependent mechanism. Given that the responses activated are critical to bacterial clearance, we propose that recognition of SLPs by TLR4 is important for recognition of the pathogen and the subsequent generation of the appropriate immune response required for bacterial clearance. This is further evidenced by our finding that a more severe disease is present in TLR4^−/−^ mice along with the absence of an antibody response to SLP, suggesting that recognition of SLPs by TLR4 may play a role in determining the outcome of infection. Furthermore, it is now well recognised that TLRs play a key role in host defence against intestinal pathogens and maintenance of tissue homeostasis in the gastrointestinal tract [34], [48]. It is of great interest that the amino acid sequence of SLP is highly variable between serogroups of *C. difficile* [49]. It is possible that these sequence differences could affect the recognition of SLPs by the innate immune system and therefore may explain why some strains of *C. difficile* cause severe infection and a high frequency of recurrence and yet others are associated with minimal clinical symptoms and pathology. While there is currently no known correlation between SLP sequence and virulence other reports suggest that variability of these surface layer proteins may be an important mechanism to escape host defence [50],[36] and warrants further investigation.

## Supporting Information

Figure S1 **SLPs induce IL-12p40 production in BMDC in the presence of polymyxin B.** DCs isolated from BALB/c mice were incubated with LPS (100 ng/mL) or SLPs (20 µg/mL) for 24 h in the presence or absence of polymyxin B. Concentrations of IL-12p40 was measured in the supernatants by ELISA. The results are the mean (±SEM) for n = 4. *** p<0.001, determined by one-way ANOVA test comparing all groups; ^+++^ p<0.001, determined by one-way ANOVA test comparing groups with and without polymyxin B.
(TIF)

Figure S2 **SLPs induce cytokine production in BMDC in a dose-dependent manner.** DCs isolated from BALB/c mice were incubated with LPS (100 ng/mL) or SLPs (5–50 µg/mL) for 24 h. Concentrations of TNFα and IL-10 were measured in the supernatants by ELISA. The results are the mean (±SEM) for n = 4. * p<0.05; *** p<0.001, determined by one-way ANOVA test comparing all groups.
(TIF)

Figure S3 **SLPs induce DC maturation in a dose-dependent manner.** DCs isolated from BALB/c mice were incubated with either LPS (100 ng/mL) or SLPs (5–50 µg/mL) for 24 h. Cells were washed and stained with antibodies specific for MHCII, CD86 or CD80 with isotype matched controls. The mean fluorescence intensity values are shown for each group. * p<0.05; *** p<0.001, determined by one-way ANOVA test comparing all groups.
(TIF)

Figure S4 **SLPs do not induce type 1 IFN production in BMDC.** DCs isolated from BALB/c mice were incubated with LPS (100 ng/mL) or SLPs (20 µg/mL) for 24 h. Concentrations of type 1 IFN was measured in the supernatants by ELISA. The results are the mean (±SEM) for n = 4. *** p<0.001, determined by one-way ANOVA test comparing all groups.
(TIF)
