# Supplementary figures and images for: A Role for TLR4 in Clostridium difficile Infection and the Recognition of Surface Layer Proteins
Source: PLoS Pathog. 2011 Jun 30;7(6):e1002076. doi: 10.1371/journal.ppat.1002076 (PMC3128122; doi:10.1371/journal.ppat.1002076)

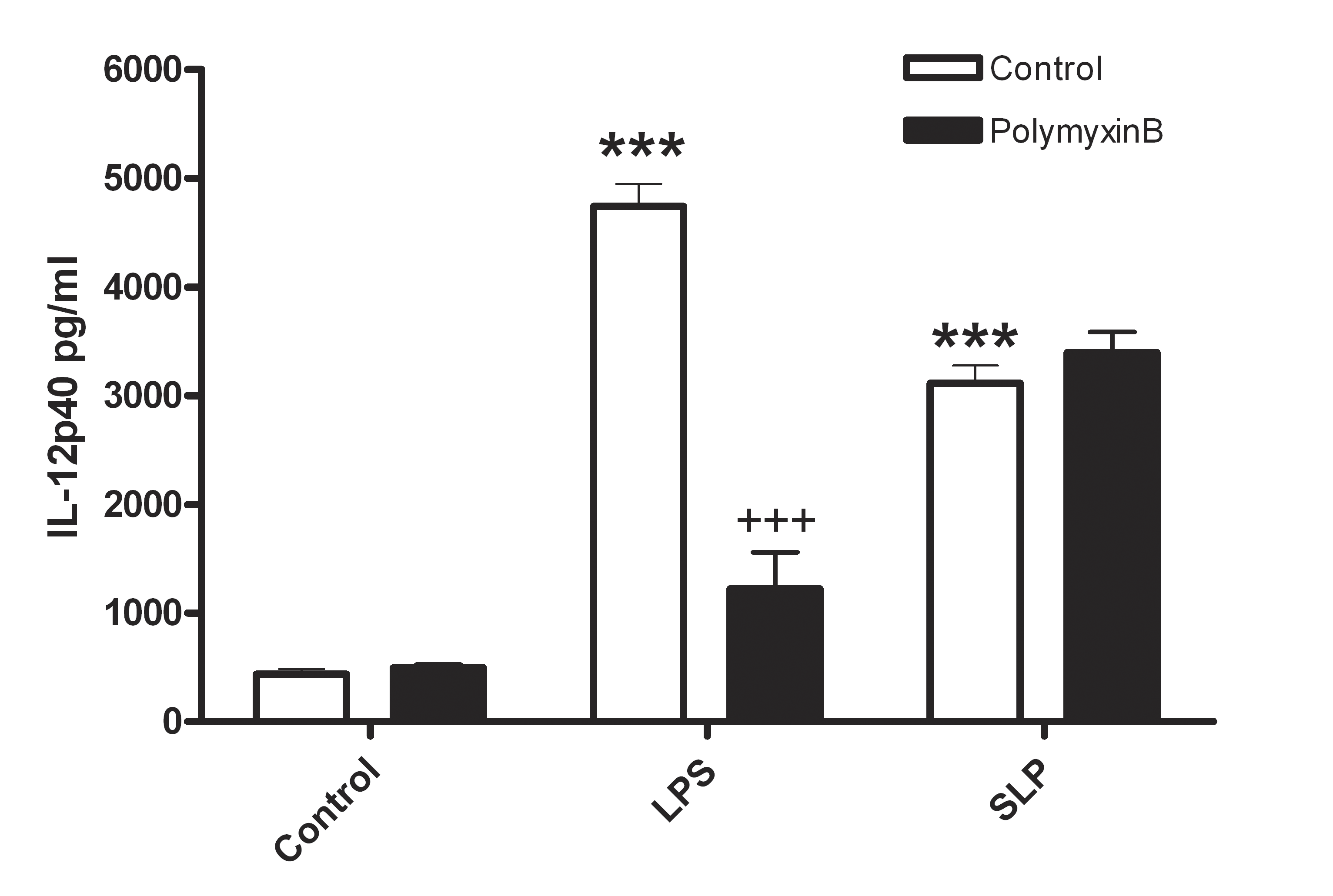

Supplement: Figure S1 — SLPs induce IL-12p40 production in BMDC in the presence of polymyxin B. DCs isolated from BALB/c mice were incubated with LPS (100 ng/mL) or SLPs (20 µg/mL) for 24 h in the presence or absence of polymyxin B. Concentrations of IL-12p40 was measured in the supernatants by ELISA. The results are the mean (±SEM) for n = 4. *** p<0.001, determined by one-way ANOVA test comparing all groups; +++ p<0.001, determined by one-way ANOVA test comparing groups with and without polymyxin B. (TIF) [file ppat.1002076.s001.tif]

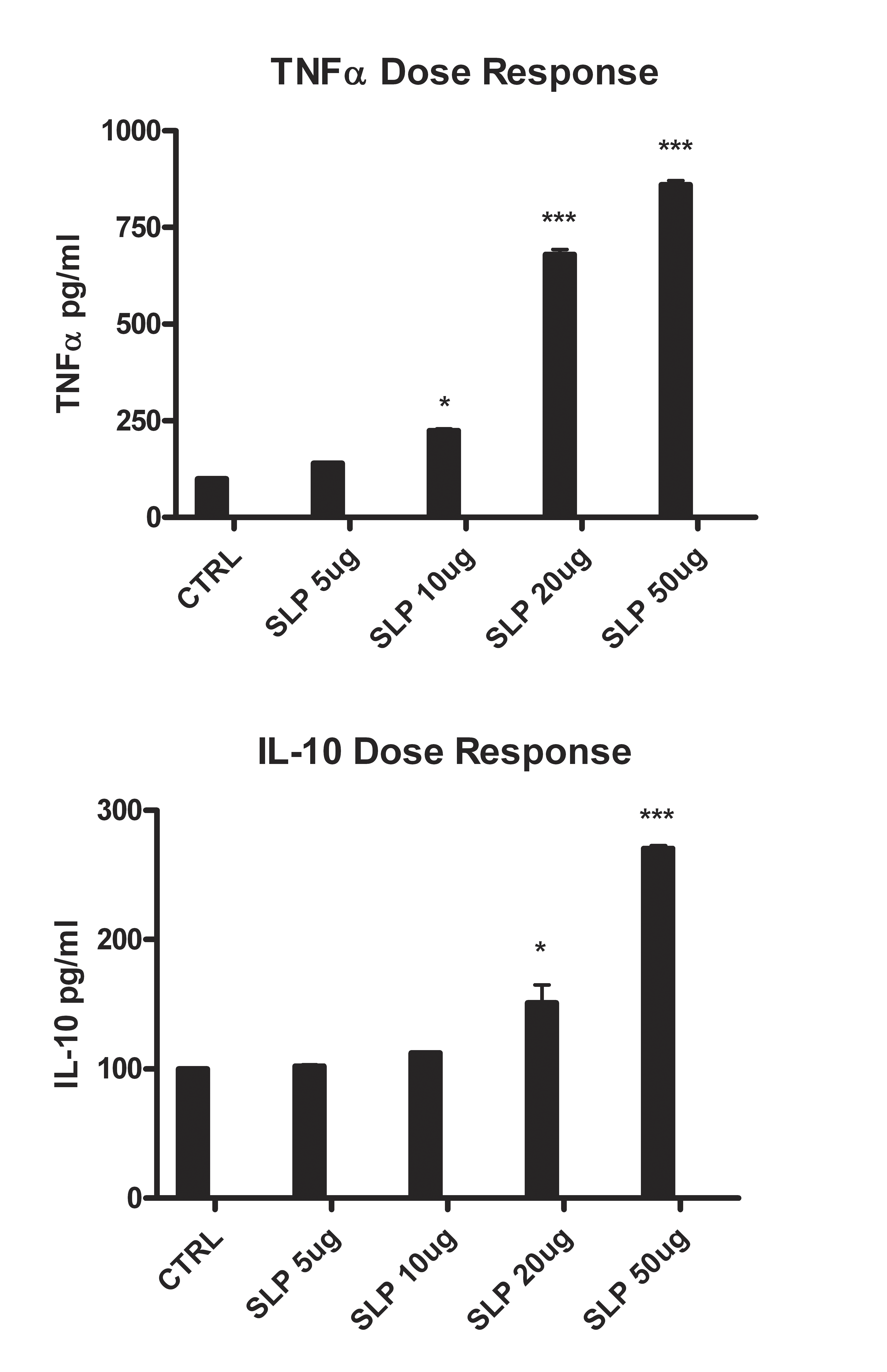

Supplement: Figure S2 — SLPs induce cytokine production in BMDC in a dose-dependent manner. DCs isolated from BALB/c mice were incubated with LPS (100 ng/mL) or SLPs (5–50 µg/mL) for 24 h. Concentrations of TNFα and IL-10 were measured in the supernatants by ELISA. The results are the mean (±SEM) for n = 4. * p<0.05; *** p<0.001, determined by one-way ANOVA test comparing all groups. (TIF) [file ppat.1002076.s002.tif]

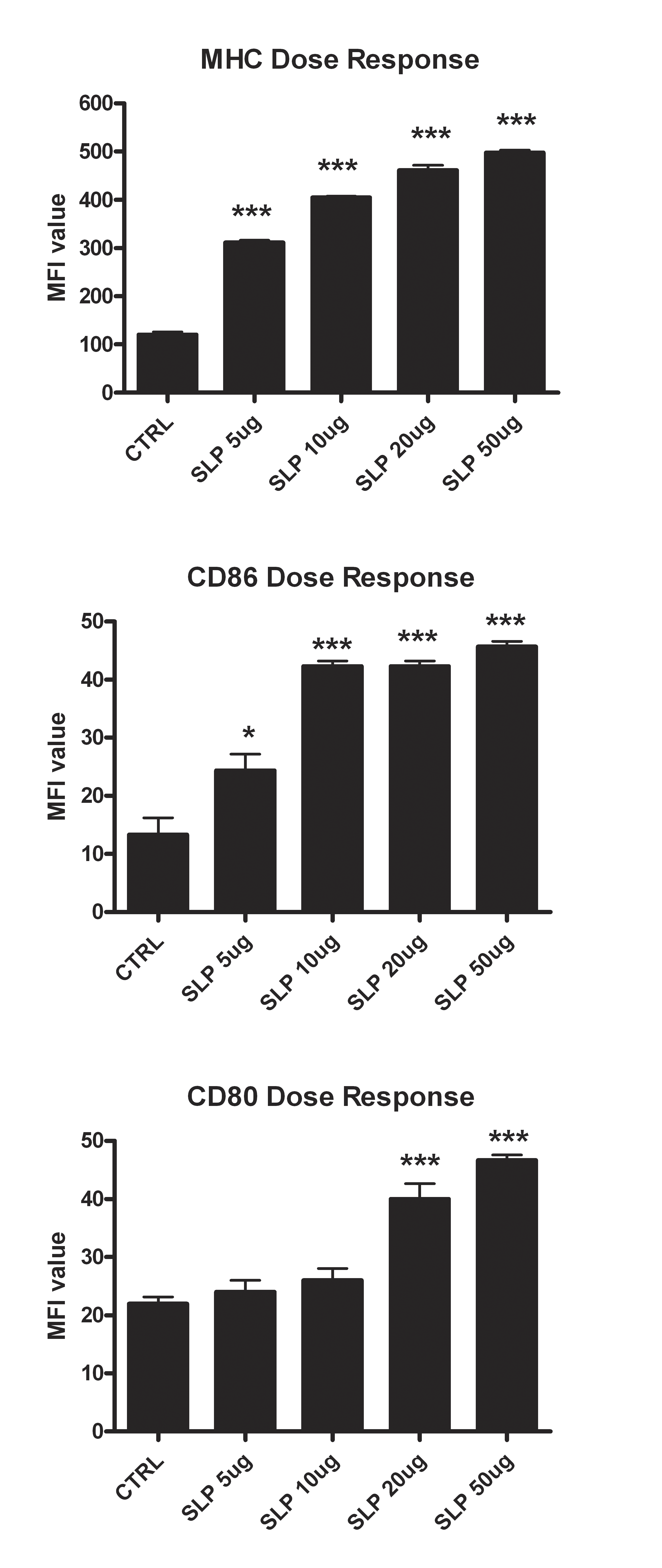

Supplement: Figure S3 — SLPs induce DC maturation in a dose-dependent manner. DCs isolated from BALB/c mice were incubated with either LPS (100 ng/mL) or SLPs (5–50 µg/mL) for 24 h. Cells were washed and stained with antibodies specific for MHCII, CD86 or CD80 with isotype matched controls. The mean fluorescence intensity values are shown for each group. * p<0.05; *** p<0.001, determined by one-way ANOVA test comparing all groups. (TIF) [file ppat.1002076.s003.tif]

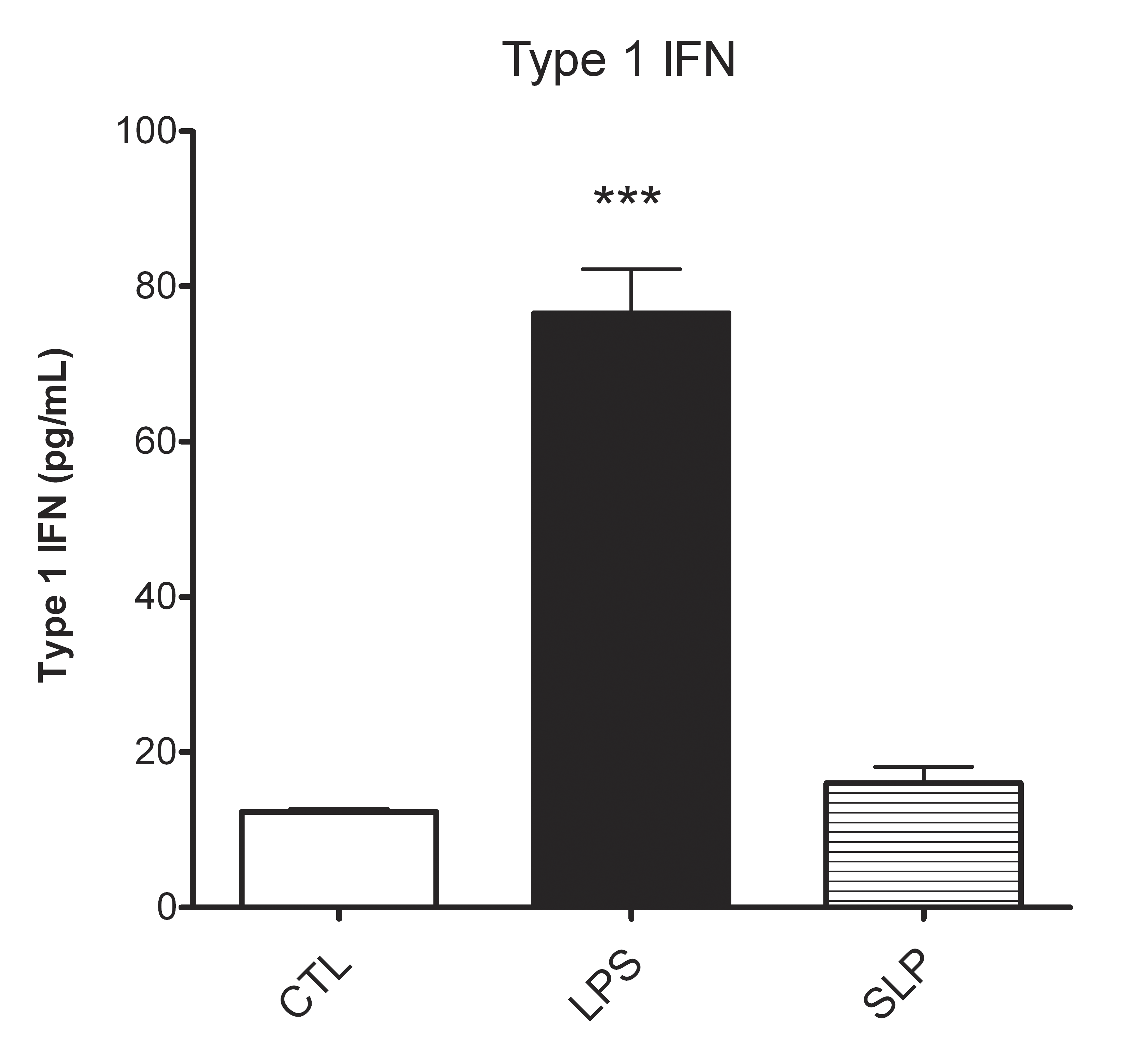

Supplement: Figure S4 — SLPs do not induce type 1 IFN production in BMDC. DCs isolated from BALB/c mice were incubated with LPS (100 ng/mL) or SLPs (20 µg/mL) for 24 h. Concentrations of type 1 IFN was measured in the supernatants by ELISA. The results are the mean (±SEM) for n = 4. *** p<0.001, determined by one-way ANOVA test comparing all groups. (TIF) [file ppat.1002076.s004.tif]
